# Physical trauma experience among school children in periurban Blantyre, Malawi

**DOI:** 10.1186/1755-7682-2-20

**Published:** 2009-07-24

**Authors:** Adamson S Muula, Humphreys E Misiri

**Affiliations:** 1 Department of Community Health, University of Malawi, College of Medicine, Blantyre, Malawi

## Abstract

**Background:**

Physical trauma is an important cause of childhood morbidity and mortality in Africa. There are however, few community-based reports on the subject on the continent. The present study was conducted to explore school children's experience of physical trauma in a disadvantaged periurban area of Blantyre, in Malawi.

**Methods:**

A cross sectional questionnaire study was carried out among school children in Ndirande-Blantyre, Malawi in 2004. Data were obtained to describe the following aspects of trauma experience: being a victim or observer of motor vehicular accidents involving pedestrians; history of falls from heights; and knowledge about road safety. Sex differences were determined for some of the variables in order to gain insights as to whether there is a difference in trauma experience between boys and girls.

**Results:**

A total of 217 school children, 99 (45.6%) boys and 118 (54.4%) girls participated in the study. Eight of them reported to have ever been hit by a motor vehicle, 87 (40.1%) had witnessed a road accident where a pedestrian had been hit and 83 (38.2%) had witnessed a pedestrian they knew having been hit by a motor vehicle. Of those that reported to have ever been hit by motor vehicle, 2 (25%) reported that they had been hospitalized as a result of injury. With regard to falling from heights, 86 reported to have ever fallen from tree, 44 of these (51.2%) were injured from the fall and 14 (16.3%) were hospitalized as a result of injury sustained from the fall. Girls were more likely to fall from trees and getting injured as compared to males (p = 0.04 for both situations). Just under half (41.9%) of the study participants were able to report the correct procedure of crossing the road despite the fact that the majority (80%) reported having been taught road safety at home or school.

**Conclusion:**

Many school children in Blantyre, Malawi have been exposed to trauma either involving themselves or someone they observed. Prevention, including education, supervision and management of trauma must receive the necessary attention they deserve in terms of resources, surveillance and impact mitigation.

## Background

Physical trauma is an important international public health problem and contributes significantly to the global burden of disease [1]. Non-fatal injuries occur among 10–30 million children and adolescents each year [2]. In low-income nations, the contribution of road trauma to overall injuries is a growing concern.

In Kenya, an epidemic of road traffic crashes resulting in a four-fold increase in the past 30 years has been witnessed [3]. An earlier study by Odero documented that driver alcohol intake was an important contributor to road accidents in that setting [4]. Ironically though in this setting, educated persons with skilled jobs were more likely to be drink-driving than non-educated persons [5], possibly as result of having greater access of disposable income coupled with limited effective intervention to prevent drink-driving.

In a study of head injuries among children presenting to hospital, Lallo and van As [6] reported that the majority of those presenting were male, and mostly having been injured after falling from heights and hit by a vehicle. Despite the growing epidemic- and especially in developing countries-, inadequate interventions are implemented to curb this pandemic, possibly due to pre-occupation with infectious diseases but also lack of adequate data to identify public health needs.

Falls from heights, especially from fruit trees, have been reported to be 'causes' of common injury in Africa [7,8]. A study by Solagberu [9] concluded that "trauma research currently command an unimportant position" in the West African region. A similar statement could be said about other parts of Africa, especially Southern Africa.

A recent systematic review of the literature on non-fatal injuries among children 5–18 years globally, found that suitable cohort studies were not available from Africa [10]. The available studies from low and middle-income nations only came from China, Taiwan and Thailand. Most of the published data on childhood injury from Africa collected data from hospital records with only a few community-based studies. While there have been a few published hospital-based studies on physical trauma in Malawi [11,12], we are unaware of any published community-based reports about children's experiences of physical trauma in the country.

## Methods

A cross sectional questionnaire study was conducted in 2004 at Ndirande Primary School (the largest public primary school in the country) among Standard 8 school children in the local language, Chichewa. The Malawi public primary school runs from Standard 1 to 8, with children enrolling in the first class at age 6 years and is offered free to all. School enrollment however, is not compulsory. Standard 8 pupils were chosen for this study as they were deemed old enough to respond to questions in the multiple choice type system. All students available in school on the day of data collection were invited to participate.

A similar approach of data collection method had been carried out among this class before in the same location of study [11,12]. The study area was a peri-urban area of Blantyre city. Ndirande is a disadvantaged community comprising both formal and informal settlements; residents do not have adequate supplies of potable water and the prevalence of diseases of poverty and poor sanitation such as intestinal helminthic infestations is high [13]. Parasitic disease rates are high due to poor sanitation and many school children are orphaned, possibly as a result of HIV and AIDS [11,12].

The school children were given a questionnaire to complete and it comprised the following issues; whether they had ever fallen from a tree, fallen from tree and got injured, ever been admitted in hospital after falling from tree (as falling from trees is a common mechanism of injury in Africa), ever witnessed a road accident that involved a motor vehicle and a pedestrian, ever witnessed a person they knew hit by motor vehicle, and whether the participant himself or herself had ever been hospitalized because of being hit by a motor vehicle. In order to obtain some idea on whether schools and parents considered road trauma and safety a priority area, we asked whether the school children had ever been taught by parents or teachers at school about road safety, and whether they had ever been tested in an examination about road safety. We also aimed to determine the knowledge level and self-assessment by the school children on how they perceived themselves as knowledgeable about road safety.

A research assistant read out each of the questions and the possible responses and allowed time for all of the children to answer a particular question before moving on to the next one. Each student then completed their responses on an individual questionnaire. This approach was done in order to enhance the response rate and ensure that the students were clear on what the question was asking them. The questionnaire were then collected and entered into an Excel spreadsheet. Analysis was done using SPSS version 9.0 to determine frequencies. The Pearson's chi-square test was carried out to determine relationships between variables and a p value of < 0.05 was considered statistically significant. Permission to conduct the study was obtained from the Ministry of Education and Human Resources.

The present study was conducted under the umbrella of the Malawi Health Equity Network (MHEN), an independent alliance of organizations and individuals established in 2000 with the aim of promoting equity and quality in health for all people in Malawi. The alliance works to improve the distribution, quality and access to health services throughout Malawi and achieves these aims by influencing government policy and practice, as well as activities of donors and civil society through advocacy, networking, research, information dissemination and budget monitoring. This research work was conducted in order to inform the debate and policy on physical trauma in the country.

## Results

### Demographic characteristics

A total of 217 school children participated in the study of which 118 (54.4%) were girls and 99 (45.6%) were boys. The age range was 12–18 years and mean was 14.5 years (standard deviation = 1.37 years). The majority of the school children 212 (97.7%) walked to school, 3 came by bus and 2 by family car. Most of the children lived with both their parents 138 (64.6%), 27 (12.4%) and 11 (5.0%) with mother only and father only respectively, while 27 (14.4%) lived with a relative not being one's parent. A total of 14 (6.5%) lived alone, with other siblings or non-relatives.

### Trauma experience

We aimed to identify what were the school children's experiences of various aspects of physical trauma. The responses regarding experience with road trauma involving a pedestrian and motor vehicle were as presented in Table 1.

**Table 1:** Experience of school children with road trauma involving pedestrian and motor vehicle

Characteristics	Yes (%)	No (%)	Missing (%)	Total
Ever been hit by motor vehicle	8 (3.7)	209 (96.3)	0 (0)	217
Ever witnessed another person hit by motor vehicle	87 (40.1)	129 (59.5)	1 (0.4)	217
Ever witnessed a person known to him/her hit by a motor vehicle	83 (38.2)	134 (61.8)	0 (0.0)	217
Ever been admitted after been hit by a motor vehicle	2 (25.0)	4 (50.0)	2 (25)	8

We also assessed sex differences of history of being hit by motor vehicle. Three (3.0%) boys and 5 (4.2%) girls reported have ever been hit. However there was no significant differences between the sexes (p = 0.638) regarding the risk of being hit by a motor vehicle.

### Experience of falling from tree

Falling from trees, especially fruit trees, has been described as a common cause of injury among children in Africa. We therefore aimed to describe whether any of the study participants had ever fallen from tree, whether they were injured from the fall and whether the injury had necessitated hospitalization. The findings are reported in Table 2 below.

**Table 2:** Reported experience from falling from tree

Experience	Yes (%)	No (%)	Total
Ever fallen from tree	86 (39.6)	131 (60.4)	217
Had injury after falling from tree	44 (51.2)	42 (48.2)	86
Was hospitalized after falling from tree	14 (16.3)	72 (83.7)	86

Of those that reported having fallen from tree, there were 32/86 boys (37.2%) and 54/86 (62.8%) girls. There was significant sex difference suggesting that more girls than boys were likely to report history of falling from tree (p = 0.044). Of those that were injured by fall from tree, 31 were males, 53 were females and the sex difference was statistically significant (p = 0.04 suggesting that more girls were likely to have been injured from falling from tree. However, regarding hospitalization as a result of fall, there were no sex differences (p = 0.44).

### Knowledge and practice about road safety

We aimed to determine importance that road safety assumes at school by asking whether the school children remembered having ever been asked a question on road safety during examinations. Some 26 (12.0%) reported having never been asked a question on road safety at examinations, 29 (34.5%) could not remember while 162 (74.6%) reported ever been exposed to an examination question on road safety. A total of 180 (82.9%) participants reported ever been taught about road safety by parents, 35 (16.1%) not ever to have had such exposure and 2 (0.9%) had missing responses.

In Malawi, vehicles are driven on the left side of the road. Pedestrians are instructed to look right first, then left and right again before crossing the road to determine if it (the road) is clear. Participants were asked which side of the road they would look first before planning to cross the road. Just under half, i.e., 91 (41.9%) had the correct answer, while 110 (50.7%) said they would look left first, 5 (2.3%) would look down and 10 (4.6%) did not know which side to look first. The majority 196 (90.3%) however reported being taught road safety at school. Just under half 46.3% (100) reported they thought they understood road signs well enough, while 32 (14.7%) thought they did not and 84 (38.7%) reported they somewhat understood and 1 (0.5%) had a missing response.

## Discussion

The present study adds to the literature on trauma in Africa by presenting data on the experience of trauma among school children in Blantyre, Malawi. Although we did not attempt to determine the distribution of causes of trauma among the study groups, just by the fact that 86% of participants reported having ever fallen from tree confirms other reports that this mechanism of injury may indeed be a common mechanism of trauma among school children in the study area.

In a hospital-based study at a referral hospital in Northern Malawi, Yu et al [14] reported that of all children presenting with injuries at Mzuzu Central Hospital, the distribution of the mechanisms of injuries was: falls (29.6%), road traffic injuries (22.0%), burns (21.4%) and poisoning (15.1%). We find that just like in our study, falls were also a common mechanism of injury. However, we had not explored burns and poisonings among our sample. Banza et al [15] found that trauma contributed 4.2% of amputations among children in Blantyre, Malawi. While congenital anomalies and other conditions predominated, this may be the case as many of the injuries sustained from trauma may have been managed without resorting to amputations.

We were however surprised to find that boys were more likely to fall from trees and get injured from the occurrence. This may result from the fact that Malawian girls, until recently, were not allowed to wear short or long pants (trousers), in preference for skirts and dresses. It is possible that such attire (dresses and skirts) may contribute to falls from trees as they may not allow flexibility/agility.

Mytton et al [10] had reported that boys were more likely to suffer from intentional injuries. However, the review by Mytton [10] had no studies from Africa. It is possible that their findings may not be applicable to an Africa setting like Malawi where the living situations may be different to the settings the original studies were conducted. We would have expected to find more boys to have been involved in falls from trees compared to girls as Malawian culture allows boys to experiment with tree climbing but girls. The reason that girls are traditionally not allowed (until recently) to be in shorts and trousers deserves further study.

The peak incidence for pedestrian injuries has been reported at 6–12 years [11,16]. With regard to pedestrian accidents, only 8 participants reported having been hit by a motor vehicle. Although the number of participants who reported to have bit hit by motor vehicle was small, the hospitalization rate at 25% is high, thus contributing significantly to morbidity. We are unable to document mortality from the trauma in this study as children who had died were not available to complete question. In addition, we did not interview parents or review hospital mortality records of nearby health facilities or referral health facilities in which severely injured school children from the study area may be treated. We also did not review police reports, or other statutory reports that may have shed further light on the prevalence of road injuries in the study area.

As trauma is a growing public health problem globally, there is need to allocate adequate resources towards research, prevention and management, including rehabilitation [17]. While there are obviously competing public health needs on the public purse, more resources to trauma are unlikely to be allocated if health practitioners, the media and other interest groups are left behind. The general public that often influences resource allocations by governments mostly does not read medical or surgical journals; medical researcher should therefore find a way of ensuring that published reports on trauma are translated for a wider, lay audience.

As this study included reported occurrences of accidents and involved only in-school children, the findings should be interpreted cautiously. Children may have misreported, just as is the case with any self-reported data. In addition, as this was a cross sectional study, we could not assign any cause-effect relationship between any of the variables. However, Malawi has a very high primary school enrollment rate of between 85–90% and therefore, the majority of children attend school. If our findings are representative of the general primary school situation, then we can postulate that injuries are an important cause of child morbidity in this setting.

## Competing interests

The authors declare that they have no competing interests.

## Authors' contributions

ASM conceived the study, designed data collection instruments, supervised data collection and participated in manuscript drafting. HEM conducted data analysis and participated in drafted manuscript drafting. All authors approved the final copy of the manuscript.
